# Source apportionment of methane escaping the subsea permafrost system in the outer Eurasian Arctic Shelf

**DOI:** 10.1073/pnas.2019672118

**Published:** 2021-03-01

**Authors:** Julia Steinbach, Henry Holmstrand, Kseniia Shcherbakova, Denis Kosmach, Volker Brüchert, Natalia Shakhova, Anatoly Salyuk, Célia J. Sapart, Denis Chernykh, Riko Noormets, Igor Semiletov, Örjan Gustafsson

**Affiliations:** ^a^Department of Environmental Science, Stockholm University, 106 91 Stockholm, Sweden;; ^b^Department of Geological Sciences, Stockholm University, 106 91 Stockholm, Sweden;; ^c^Bolin Centre for Climate Research, Stockholm University, 106 91 Stockholm, Sweden;; ^d^V.I. Il’ichev Pacific Oceanological Institute, Far Eastern Branch of the Russian Academy of Sciences, Vladivostok, 690041, Russia;; ^e^Institute of Ecology, Higher School of Economics, Moscow, 101000, Russia;; ^f^International Arctic Research Center, University of Alaska Fairbanks, Fairbanks, AK 99775;; ^g^Scientific Centre Moscow State University-Geophysics, Moscow, 119991, Russia;; ^h^Laboratoire de Glaciologie, Université Libre de Bruxelles, 1050 Brussels, Belgium;; ^i^Institute for Marine and Atmospheric Research, Utrecht University, 3584 CC Utrecht, The Netherlands;; ^j^Department of Arctic Geology, University Centre Svalbard, Longyearbyen, N-9171, Norway

**Keywords:** methane, isotopes/radiocarbon, Arctic, carbon cycle/climate change, subsea permafrost

## Abstract

The East Siberian Arctic Shelf holds large amounts of inundated carbon and methane (CH_4_). Holocene warming by overlying seawater, recently fortified by anthropogenic warming, has caused thawing of the underlying subsea permafrost. Despite extensive observations of elevated seawater CH_4_ in the past decades, relative contributions from different subsea compartments such as early diagenesis, subsea permafrost, methane hydrates, and underlying thermogenic/ free gas to these methane releases remain elusive. Dissolved methane concentrations observed in the Laptev Sea ranged from 3 to 1,500 nM (median 151 nM; oversaturation by ∼3,800%). Methane stable isotopic composition showed strong vertical and horizontal gradients with source signatures for two seepage areas of δ^13^C-CH_4_ = (−42.6 ± 0.5)/(−55.0 ± 0.5) ‰ and δD-CH_4_ = (−136.8 ± 8.0)/(−158.1 ± 5.5) ‰, suggesting a thermogenic/natural gas source. Increasingly enriched δ^13^C-CH_4_ and δD-CH_4_ at distance from the seeps indicated methane oxidation. The Δ^14^C-CH_4_ signal was strongly depleted (i.e., old) near the seeps (−993 ± 19/−1050 ± 89‰). Hence, all three isotope systems are consistent with methane release from an old, deep, and likely thermogenic pool to the outer Laptev Sea. This knowledge of what subsea sources are contributing to the observed methane release is a prerequisite to predictions on how these emissions will increase over coming decades and centuries.

## Significance

Extensive release of methane from sediments of the world’s largest continental shelf, the East Siberian Arctic Ocean (ESAO), is one of the few Earth system processes that can cause a net transfer of carbon from land/ocean to the atmosphere and thus amplify global warming on the timescale of this century. An important gap in our current knowledge concerns the contributions of different subsea pools to the observed methane releases. This knowledge is a prerequisite to robust predictions on how these releases will develop in the future. Triple-isotope–based fingerprinting of the origin of the highly elevated ESAO methane levels points to a limited contribution from shallow microbial sources and instead a dominating contribution from a deep thermogenic pool.

The East Siberian Arctic Shelf (ESAS) is the world’s largest and shallowest shelf sea system, formed through inundation of northeast Siberia during sea level transgression in the early Holocene. The ESAS holds substantial but poorly constrained amounts of organic carbon and methane (CH_4_). These carbon/methane stores are contained in unknown partitions as gas hydrates, unfrozen sediment, subsea permafrost, gas pockets within and below the subsea permafrost, and as underlying thermogenic gas (1–3). Methane release to the atmosphere from these compartments could potentially have significant effects on the global climate (4, 5), yet there are large uncertainties regarding the size and the vulnerability toward remobilization of these inaccessible and elusive subsea carbon/methane pools. Conceptual development and modeling have predicted that warming of the ESAS system by a combination of geothermal heat and climate-driven Holocene heat flux from overlying seawater, recently further enhanced by Anthropocene warming, may lead to thawing of subsea permafrost (6, 7). Subsea permafrost drilling in the Laptev Sea, in part at the same sites as 30 y ago, has recently confirmed that the subsea permafrost has indeed come near the point of thawing (8). In addition to mobilization of the carbon/methane stored within the subsea permafrost, its degradation can also lead to the formation of pathways for gaseous methane from underlying reservoirs, allowing further methane release to the overlying water column (3, 9).

Near-annual ship-based expeditions to the ESAS over the past two decades have documented widespread seep locations with extensive methane releases to the water column (3, 10). Methane levels are often found to be 10 to 100 times higher than the atmospheric equilibrium and are particularly elevated in areas of strong ebullition from subsea gas seeps (“methane hotspots”). Similarly, elevated dissolved methane concentrations in bottom waters appear to be spatially related to the thermal state of subsea permafrost as deduced from modeling results and/or geophysical surveys (7, 9). Currently, we lack critical knowledge on the quantitative or even relative contributions of the different subsea pools to the observed methane release, a prerequisite for robust predictions on how these releases will develop. An important distinction needs to be made between pools that release methane gradually, such as methane produced microbially in shallow sediments during early diagenesis or in thawing subsea permafrost, versus pools with preformed methane that may release more abruptly once pathways are available, such as from disintegrating methane hydrates and pools of thermogenic (natural) gas below the subsea permafrost. Multidimensional isotope analysis offers a useful means to disentangle the relative importance of these different subsea sources of methane to the ESAS: Stable isotope data (δ^13^C-CH_4_ and δD-CH_4_) provide useful information on methane formation and removal pathways, and the radiocarbon content of methane (Δ^14^C-CH_4_) helps to determine the age and methane source reservoir (see *SI Appendix*, text S1 for details on these isotope systematics and typical isotopic signatures for the ESAS subsea system).

Here, we present triple-isotope–based source apportionment of methane conducted as part of the Swedish–Russian–US investigation of carbon–climate–cryosphere interactions in the East Siberian Arctic Ocean (SWERUS-C3) program. To this end, the distribution of dissolved methane, its stable carbon and hydrogen isotope composition, as well as natural radiocarbon abundance signature, were investigated with a focus on the isotopic fingerprint of methane escaping the seabed to pinpoint the subsea sources of elevated methane in the outer Laptev Sea.

## Results and Discussion

### Study Area and Geophysical Surveying.

The SWERUS-C3 expedition with the Swedish icebreaker (IB) *Oden* in 2014 primarily targeted the outer ESAS (water depth >50 m) because of the following reasons: 1) this area is relatively understudied compared to the inner to midshelf areas and 2) the underlying permafrost of this region is more degraded/discontinuous due to its longer exposure to warming overlying seawater since inundation (6, 7).

**Fig. 1.**
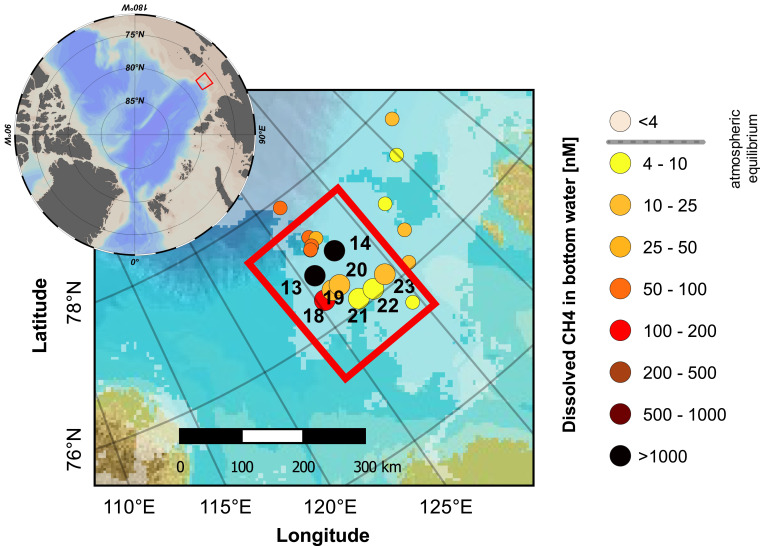
The study area and sampling locations for CH_4_ concentration and isotopes. The colored dots show the locations of samples taken in the northern Laptev Sea during the SWERUS-C3 2014 expedition, with the colors representing concentration of dissolved CH_4_ in bottom water (sampling depth ca. 5 m above bottom). The subset of sampling stations that were used for this study is highlighted with larger symbols and station numbers.

The selection of detailed study areas and sampling locations was guided both by earlier studies (10, 11) and by continuous geophysical sounding for seafloor seeps, sites of bubble ebullition in the water column, and other potentially gas-related features in the sediments (see *Materials and Methods*). A larger methane seep area in the outer Laptev Sea was chosen for in-depth methane source apportionment using the triple-isotope approach. Sampling focused on an area located between 125 and 130°E and 76 and 77°N in water depths of 46 to 72 m (Fig. 1). Gas blankings detected by the acoustic subbottom profiler indicated high gas saturation in the sediments in this area (*SI Appendix*, Fig. S2). A total of 160 gas seeps extending through the water column were recorded by the midwater sonar. These methane venting areas were discovered and documented during earlier expeditions in 2011, 2012, and 2013 for elevated methane concentrations and occurrence of bubbles throughout the water column (11). Sampling in the study area was performed from July 18 to 22, 2014. During this period, the water was completely ice free, and the weather conditions were calm with stable wind speeds less than 6 m/s and low wave heights of <0.5 m.

**Fig. 2.**
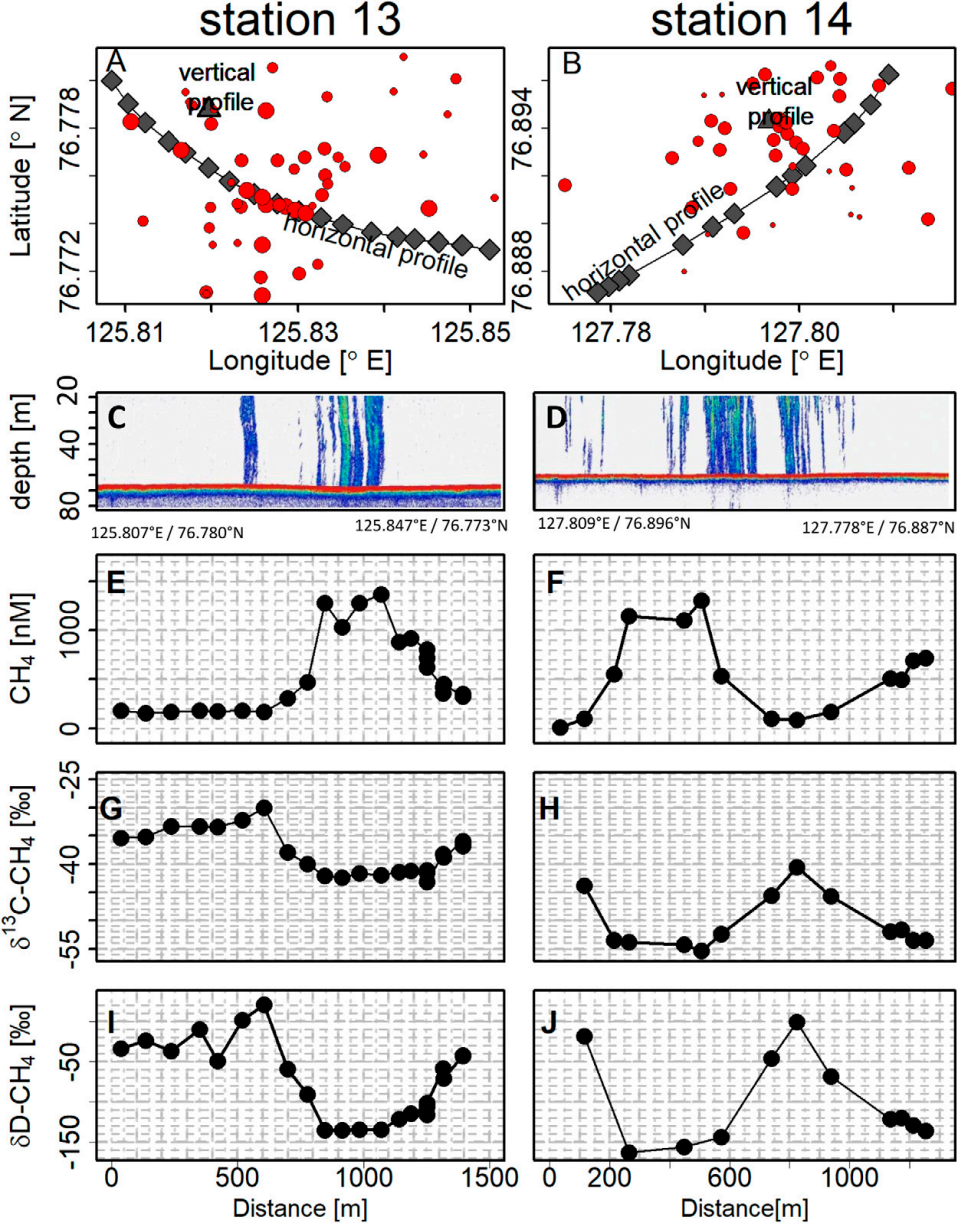
The horizontal bottom water profiles and distribution of gas seeps. Data from bottom waters and distribution of gas seeps around hotspots at stations 13 (left column) and 14 (right column). Row 1 (*A* and *B*) shows sampling locations for vertical (triangles) and horizontal (diamonds) profiles and locations of gas seeps (red dots) as identified by midwater echosounder. The dot size is proportional to observed seep size by the echosounder (compare *SI Appendix*, Fig. S2). (*C*–*J*) Horizontal data, plotted versus distance from the start of the transect (see start and end coordinates in the respective column). Row 2 (*C* and *D*) shows water column echosounder data along the drift, row 3 (*E* and *F*) shows concentration of dissolved CH_4_, row 4 (*G* and *H*) shows δ^13^C-CH_4_, and row 5 (*I* and *J*) shows δD-CH_4_ from Niskin bottles. Average sampling depth is ∼5 m above sea bottom; water depths are 68 m for station 13 and 61 m for station 14.

### Vertical Water Column Profiles.

**Fig. 3.**
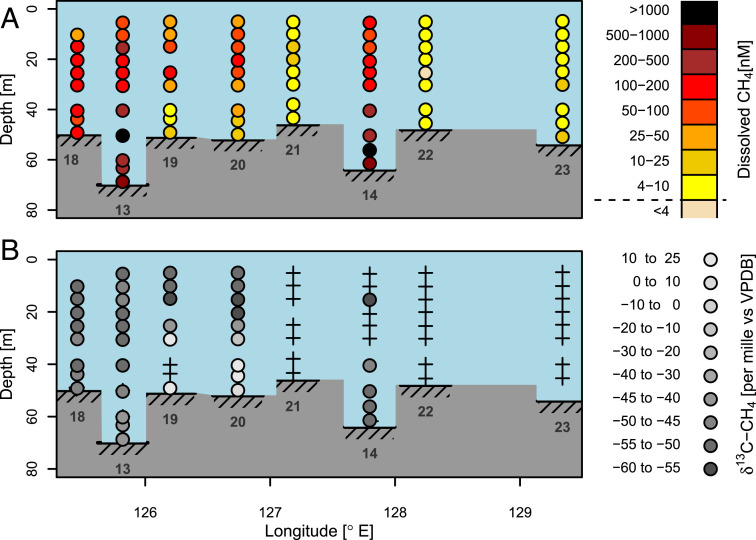
Vertical water column profiles. (*A*) Dissolved CH_4_ concentration. (*B*) δ^13^C-CH_4_. The dashed line at the concentration value of 4 nM in the legend of *A* indicates the dissolved CH_4_ concentration corresponding to equilibrium with the overlying atmosphere. The crosses in *B* illustrate locations where samples have been taken but no δ^13^C-CH_4_ analysis was performed. The inserted numbers below each profile denote the station number.

A total of eight sampling stations were chosen for detailed water column investigations (Fig. 1): two “ebullition hotspots” (high methane concentration and strong ebullition throughout the entire water column; stations 13 and 14); three stations surrounded by smaller seeps with less intensive ebullition (stations 18 through 20); and three stations where no seep features or ebullition were observed (stations 21 through 23). Enhanced methane concentrations were found in the water column profiles of all eight stations, with overall concentrations ranging from 3 to 1,511 nM and a median concentration of 151 nM, corresponding to an oversaturation of ∼3,800% relative to the atmospheric equilibrium concentrations of ∼4 nM (Fig. 3*A*). The highest concentrations were observed at the hotspot stations 13 and 14 (median values of 314 and 218 nM, respectively). At these two stations, the maximum dissolved methane concentrations (1,367 and 1,422 nM) occurred close to the bottom, consistent with the presence of a subsea source. At the stations without or with only small seeps in the direct vicinity, methane concentrations were lower but still well above equilibrium levels (maxima of 100 to 140 nM at stations 18 through 20, 16 nM at stations 21 through 23). At the stations without seeps (21 through 23), the concentration maxima occurred higher up in the water column, just beneath the pycnocline, which typically was located at 25- to 30-m water depth. However, dissolved methane concentrations at these more distant stations also were consistently above the equilibrium values, even near the water surface at 5-m depth, with levels varying between 6 and 72 nM.

The δ^13^C-CH_4_ composition in the water column ranged from −56.5 ‰ to ±22.2 ‰ (Fig. 3*B*). For most stations, the lightest isotope signature corresponded to the (locally) highest concentration. This relationship was most pronounced at stations 13 and 14, which were closest to the strongest seep sources. The δD-CH_4_ data (measured on selected samples) showed similar patterns as δ^13^C-CH_4._ The δD-CH_4_ values ranged from −167.8 to −117.3‰ for station 13, from −82.2 to −66.6 ‰ for station 14, and from −152.6 to −48.3 ‰ for station 18.

### Horizontal Bottom Water Profiles.

Bubble plumes identified by the midwater echosounder strongly suggested ebullition from the sediment seeps as the predominant source of methane to the water column. Ascending bubbles were also observed visually at the sea surface at some stations. A more detailed investigation of the dissolved concentration and isotopic signature of methane was thus performed across two regions with distinct bubble plumes (stations 13 and 14). Near-bottom seawater was collected in Niskin bottles while the sampling rosette was allowed to drift horizontally across the acoustically identified seep area (“drift casts” as described in *Materials and Methods*). High dissolved methane concentrations in the bottom waters coincided with nearby locations of acoustic bubble signals, and increasingly depleted δ^13^C-CH_4_ and δD-CH_4_ isotope composition was observed toward the maxima in methane concentrations (Fig. 2). Peak CH_4_ concentrations closest to the seep reached 1,300 to 1,400 nM, with concentrations at a distance of 300 to 500 m from the main seep, still on the order of 150 to 500 nM. Isotopic signatures at the CH_4_ concentration maxima were δ^13^C-CH_4_ = −42.0 ‰/−55.2 ‰ and δD-CH_4_ = −135.2 ‰/−165.2 ‰ for stations 13 and 14, respectively. The isotopic composition corresponding to the lower CH_4_ concentrations further away from the seep center were more enriched, with δ^13^C-CH_4_ = −34.9 ± 2.5 ‰/−43.9 ± 2.4 ‰ and δD-CH_4_ = −28.9 ± 26.6 ‰/−33.6 ± 30.1 ‰ for stations 13 and 14, respectively.

### Natural Abundance Radiocarbon Signal of CH_4_.

Samples from the two major seep stations (stations 13 and 14, 3 different depths) and one station without apparent seeps (station 19, one depth) had sufficiently high levels of radiocarbon for the determination of Δ^14^C-CH_4._ The radiocarbon signature of the sample from the station without seeps was Δ^14^C-CH_4_ = −478 ± 29 ‰. Samples from the seep stations were significantly more depleted in Δ^14^C-CH_4_ (= older): They ranged from Δ^14^C-CH_4_ = (−749 ± 18) ‰ to Δ^14^C-CH_4_ = (−949 ± 28) ‰ for station 13 and from Δ^14^C-CH_4_ = (−815 ± 29) ‰ to Δ^14^C-CH_4_ < −1,000 for station 14. The oldest signals for both systems were recorded in the methane collected closest to the sediment source.

### Processes Affecting Spatial Distribution of Water Column Methane.

Methane concentrations and isotopic signatures in ESAS seawater are generally influenced by a mixture of sources and degradation processes (*SI Appendix*, text S1 and Fig. S1). The presence of an ebullition-transported subsea source at seep sites is clearly visible from the distribution of methane concentrations in both vertical and horizontal profiles, as well as from the sonar-documented ebullition. Ebullition can effectively transport CH_4_ through the pycnocline to the water surface. Vertical profiles closest to the seeps show concentration maxima at the bottom and midwater maxima for most of the other stations. The latter may reflect accumulation of upward-diffusing methane at the pycnocline.

The vertical distribution of dissolved methane likely reflects continuous dissolution of methane from the bubbles into the water column. The vertical concentration profiles and the timing of CH_4_ venting to the atmosphere are also affected by water column mixing and changes in meteorological conditions (12). The relative magnitude of these ebullition and dissolution vectors is, however, difficult to assess without quantitative measurements of the amount of bubbles and their methane content. The presence of bubbles can also influence the observed isotopic signatures; dissolved methane and gaseous methane would be altered to different degrees by methane oxidation, as that process only affects CH_4_ in its dissolved form.

Methane oxidation (13) is a likely explanation for the extremely enriched (positive) δ^13^C-CH_4_ signatures at stations 19 and 20 (up to +16.1‰ and +22.1‰, respectively). These values correlate with relatively lower CH_4_ concentrations—consistent with the oxidation of a large fraction of the methane, leaving the residual dissolved pool heavily enriched. Such highly enriched stable isotopic signatures due to oxidation are unusual but have been reported elsewhere (14, 15). This strong enrichment implies long residence times, which may be achieved in this system by residing in the surface sediments (anaerobic oxidation) or in the water column (aerobic oxidation). This altered methane is thus not traceable to a specific (local) source but is the product of a series of mixing/alteration processes during several-year-long circulation on the ESAS. The presence of such a degradation process complicates stable-isotope–based source apportionment of dissolved methane (Δ^14^C is, per definition, unaffected by isotope fractionation; see *SI Appendix*, text S1). Therefore, our isotopic fingerprinting of the sources of the bottom-escaping methane focuses on the samples closest to the seeps.

A Keeling plot approach (16, 17) is a common way to handle such effects and to constrain sources; this was also used here to fingerprint the dominant source(s) of CH_4_ from the subsea system. The basic assumption for this method is the presence of a single source mixed with a background reservoir—a simplified approach whose use for a complex system as here needs to be assessed carefully (18, 19). The steep gradients in methane concentration and isotopic signatures in the vicinity of the sediment seeps provide a promising setting for a successful application of the Keeling plot approach. Methane emanating from the sediment was treated as a uniform “sediment source” pool that mixed with the “background” water column methane. Influences of methane degradation and mixing with water column sources were assumed to be negligible in close proximity to the subsea seep source. Partial dissolution of bubbles into the water column and their effect on the isotopic signature are difficult to quantify but are likely to be less significant closer to the seafloor.

### Stable Isotope Constraints on the Sedimentary Methane Source.

**Fig. 4.**
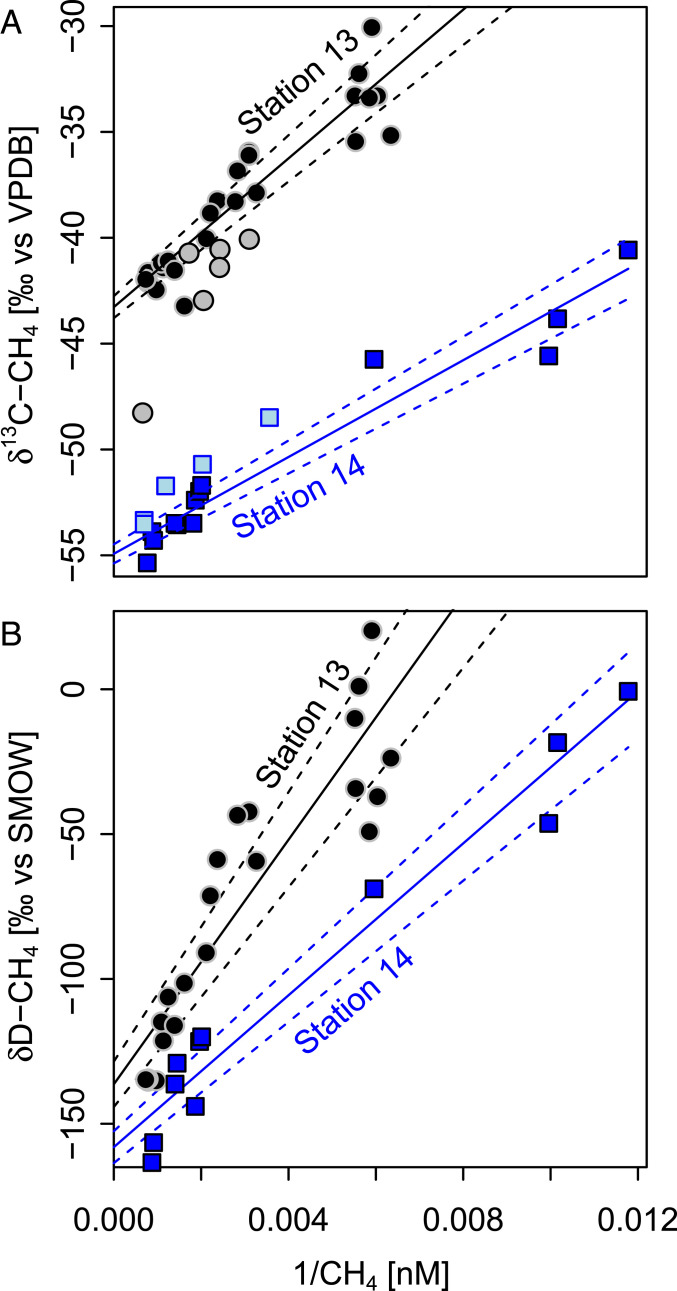
The Keeling plots for quantitative source constraint. Keeling plots for δ^13^C-CH_4_ (*A*) and δD-CH_4_ (*B*) for the two strongest seep stations (dots for station 13, squares for station 14). The black and dark blue symbols show data from horizontal bottom water profiles, and straight lines indicate Keeling plot fits for these data with dashed lines indicating error of the fit. The gray and light blue symbols in *A* show the data used for Keeling plot fits of the corresponding vertical water column profile.

Keeling plots of the lower part of the water column profiles (Fig. 4 and *SI Appendix*, Fig. S3) suggest a δ^13^C-CH_4_ source signature of −49.0 ± 2.0 ‰ (data below 50 m, *R*^2^ = 0.70) at station 13, and −54.3 ± 0.4. ‰ (data below 30 m, *R*^2^ = 0.95) at station 14. The good linear fits of the Keeling plots lend credence to the applicability of this approach for constraining the isotope fingerprint of the methane escaping the seafloor. These source signatures are more enriched than typical methane in marine near-surface sediments and thus indicate a thermogenic origin of methane from the seep source (*SI Appendix*, text S1). Bottom water δD-CH_4_ at these stations is also consistent with this assessment.

For the upper part of the water column and for the stations further away from the strongest seeps, the Keeling plots were inconclusive (*SI Appendix*, Fig. S3). This suggests that the assumption of a two-component mixture (dominant sediment source versus seawater background) is not valid for mid and surface water further away from the seeps, either because the influence of the subsea source was not strong enough compared to other methane sources or because the isotopic composition of the sediment-derived methane has been modified by oxidation after mixing with seawater.

The horizontal bottom water profiles across the seeps, like the lower part of the vertical profiles, showed clear correlations between CH_4_ concentration and δ^13^C/δD-CH_4_. Here, Keeling plots (Fig. 4) yielded well-constrained source signatures of δ^13^C-CH_4_ = −42.6 ± 0.5 ‰ (for station 13, *R*^2^ = 0.88) and −55.0 ± 0.5 ‰ (for station 14, *R*^2^ = 0.94) and δD-CH_4_ = −136.8 ± 8.0 ‰ (station 13, *R*^2^ = 0.81) and −158.1 ± 5.5 ‰ (station 14, *R*^2^ = 0.95), respectively. The δ^13^C-CH_4_ source signatures were comparable to those determined from the water column profile data and are, together with δD-CH_4,_ consistent with a thermogenic/natural gas source with minor contributions from microbial sources. Samples with lower concentrations, taken further away from the seep centers, deviate significantly from the linear fit in the Keeling plots for the horizontal bottom water profiles. At these locations, methane is apparently influenced by processes other than mixing between the sediment source and the surrounding waters. The slopes of δD-CH_4_ versus δ^13^C-CH_4_ for these samples indicated that their enriched isotopic values are indeed likely due to CH_4_ oxidation (*SI Appendix*, Fig. S4). Nevertheless, the correlations in the Keeling plots are significant for samples in closer vicinity to the sources. In addition, using different subsets of the horizontal profile data for the calculation of the Keeling line (i.e., including varying numbers of datapoints further away from the seep center) showed no significant changes in the *y*-intercept. Hence, the Keeling plot approach seems to give robust results for the signature of the seep source.

### Radiocarbon Constraints on the Sedimentary Methane Source.

**Fig. 5.**
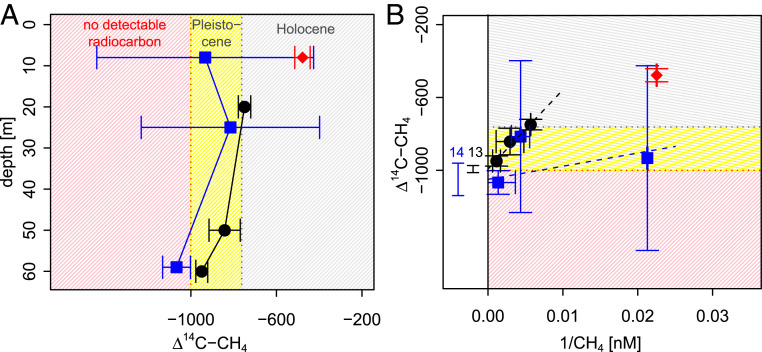
The radiocarbon content of CH_4_ in water column profiles. The black circles show data from station 13, blue squares from station 14, and orange diamonds from station 19 (water data taken in 8 m depth from a station without seeps, added for comparison). (*A*) Depth profiles of Δ^14^C-CH_4_ and (*B*) Keeling plots for Δ^14^C-CH_4_ at the seep stations, with the bars on the left side of the plot indicating the intercept of the Keeling fit and its total error (*SI Appendix*, text S2).

The Δ^14^C-CH_4_ signal is not influenced by methane oxidation or other fractionation processes (*SI Appendix*, text S1) and thus provides an unbiased and independent source constraint. The Δ^14^C-CH_4_ water column profiles show a trend of increasing CH_4_ radiocarbon age with depth (Fig. 5*A*), which is consistent with an old methane source emitted from the sediment that is mixed with (on average) younger methane in shallower depths. Keeling plots for the radiocarbon data gave source signatures of Δ^14^C-CH_4_ = −993 ± 19 ‰ for station 13 (*R*^2^ = 0.97) and Δ^14^C-CH_4_ = −1,050 ± 89 ‰ for station 14 (*R*^2^ = 0.24) (Fig. 5*B*). With Δ^14^C-CH_4_ = −1,000 being the detection limit for radiocarbon (corresponding to radiocarbon ages of >60 kyr), this points to a sedimentary methane source that is either thermogenic (containing no detectable radiocarbon) or microbial and very old (at least mid-Pleistocene aged).

### Constraints on the Sedimentary Methane Source from Triple Isotopes and Other System Parameters.

**Fig. 6.**
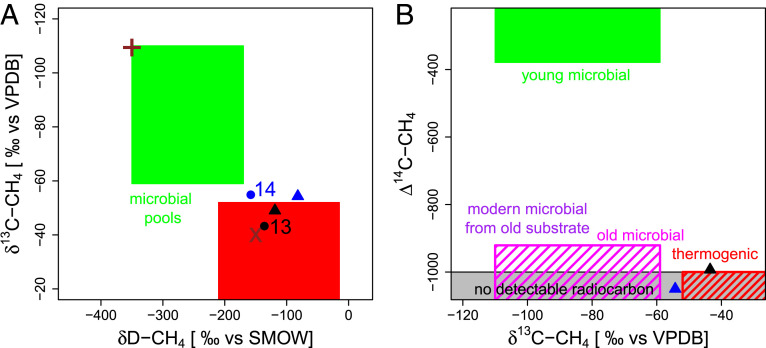
Triple-isotope fingerprints in the context of potential ESAS source pools. (*A*) Stable-isotope signatures and (*B*) radiocarbon plotted against δ^13^C-CH_4_. The data points shown here are the result of source extrapolation from Keeling plot for stations 13 (black) and 14 (blue). The triangles represent the Keeling plot intercepts of water column profile data, and spheres represent those from horizontal bottom water profiles. Earlier stable-isotope data from Sapart et al. [inner Laptev Sea sediment, + symbol (18)] and Cramer and Franke [outer Laptev Sea, x symbol; (24)] are added for comparison. The chosen isotopic signatures for the endmember pools are summarized and explained in *SI Appendix*, text S1.

The results of the combined triple-isotope source apportionment are summarized in Fig. 6. To facilitate interpretation, we grouped the potential subsea sources of CH_4_ to the ESAS bottom waters into four different endmember pools based on putative formation mechanism and anticipated isotopic fingerprints (see Fig. 6 and *SI Appendix*, text S1 for details). A literature review indicated a significant range of isotope compositions for these pools (*SI Appendix*, text S1). The depiction of the pools in Fig. 6 shows the range of discrete values that—based on our review of available studies—best reflects the settings in the ESAS.

The results consistently point to the presence of an old, predominantly radiocarbon-depleted source of thermogenic origin or a mixture of thermogenic and old microbial sources. The stable isotope source signatures for station 13 fall into a range typical for thermogenic/natural gas origin, whereas the slightly more depleted signal at station 14 indicates additional input from another, potentially microbial origin (Fig. 6*A*). Both the old radiocarbon signatures (Fig. 6*B*) and the ebullitive nature of methane at these stations, characterized by abrupt releases and strong spatial gradients in the water column, suggest a deep, advective methane pool as an important contributor to the observed water column methane signal.

To test the triple-isotope–based source constraint of a predominantly thermogenic source, other relevant ancillary data were also considered: First, our seawater samples were checked for their contents of higher hydrocarbons (here, explicitly ethane) as these are often found in association with thermally created methane, at least in direct connection to thermogenic source compartments. No detectable ethane levels were found in any of the samples. However, this is understandable from the composition of this system where the thermogenic gas is formed at a depth of several kilometers and has been subject to migration through the sedimentary drape, including attenuation by subsea permafrost, before a portion of it is finally released to the overlying seawater. The long timescale of this transport is likely the reason why higher hydrocarbons have been microbially degraded below the detection limit before reaching the water column. Preferential biodegradation of C2+ hydrocarbons has been described by several studies (20–23); additionally, initial concentrations of higher hydrocarbons in the source pool are already expected to be significantly lower than those of methane (24).

The presence of a thermogenic source pool beneath our study area is consistent with results by Cramer and Franke (24), based on their observations of hydrocarbon concentrations and their δ^13^C in adsorbed gases in the sediment. The existence of pathways to transport methane from these deep sources to the water column in our study area is also consistent with recent seismic data (25), which show ∼500-m wide gas conduits in the sediment, correlating with a fault zone and cuts through the Neogene succession to the basement. Further support for a migratory inflow of petroleum hydrocarbons from below is also given by a recent biomarker study in the studied seep area (26): Excess amounts of two molecular markers typical of a petrogenic source have been found in the surface sediment of the studied seep area, with significant differences between the seepage area and the “background areas” without apparent seeps. Taken together, the triple isotopes and these other ancillary data are consistent with a deep thermogenic source of methane.

There are a few earlier studies that have provided an important motivation for the current triple-isotope–based study. Cramer and Franke (24) suggested based on single-isotope data that thermally generated gas from petroleum rocks was a key source of methane to the Laptev Sea sediment. Indications of leakage from this source to the water column were seen in δ^13^C-CH_4_ signatures at a few stations, in contrast to microbial-typical signatures dominating in the inner shelf. Based on δ^13^C-CH_4_ and δD-CH_4_ isotope data, Sapart et al. (18) suggested an old, Pleistocene CH_4_ source, but of microbial origin, for the innermost SE Laptev Sea. Their study also reported more enriched stable isotopic signatures in the water column near our study region in the outer Laptev Sea and attributed these either to an additional thermogenic source or to substantial oxidation of CH_4_ from the deep sediment, partly in line with the interpretations deduced from the triple-isotope result of the present study that is pointing consistently to a deep thermogenic source at the outer Laptev Sea seep sites.

Mobilization of methane originating from an old sedimentary source was also indicated on the other side of the Arctic Ocean at the US Beaufort Shelf. Using Δ^14^C-CH_4_ data_,_ Sparrow et al. (19) estimated significant contributions (45 to 86%) of ancient sources of methane found in waters, yet much lower dissolved methane concentrations are observed on the American shelfs than what is found on the ESAS.

The present study demonstrates the strengths of the triple-isotope approach in contrast to earlier single- or dual-isotope studies. Given the large ranges in the stable isotopes of putative methane sources, both δ^13^C-CH_4_ and δD-CH_4_ are needed to narrow down potential CH_4_ origins. Since stable isotope signatures are sensitive to fractionation processes, such as by aerobic and anaerobic CH_4_ oxidation, a dual stable-isotope source apportionment is difficult for locations further away from seep sites or other strong sources. This study also shows that the generic stable-isotope ranges (*SI Appendix*, Fig. S1) for different methane origins/formation processes are not sufficient for quantitative source apportionment: Improved observation-based knowledge of locally relevant processes and end-member signatures is necessary to make further headway in our understanding of the heterogeneous ESAS system. Radiocarbon data of CH_4_ added a powerful constraint. However, when it is used as the sole tracer, it is also not enough for unambiguous source apportionment since it cannot distinguish old Pleistocene methane trapped in permafrost from recently formed methane from an old radiocarbon-depleted carbon source. The combination of the two stable-isotope systems with the radiocarbon signals gives the greatly improved constraints and apportionment of the methane sources.

Taken together, the triple-isotope data presented here, in combination with other system data and indications from earlier studies, suggest that deep thermogenic reservoirs are key sources of the elevated methane concentrations in the outer Laptev Sea. This finding is essential in several ways: The occurrence of elevated levels of radiocarbon-depleted methane in the water column may be an indication of thawing subsea permafrost in the study area (see also ref. 8). The triple-isotope fingerprinting suggests, however, that methane may not primarily originate directly from the subsea permafrost; the continuous leakage of an old geological reservoir to the water column suggests the existence of perforations in the subsea permafrost, serving as conduits of deeper methane to gas-charged shallow sediments. Second, the finding that methane is released from a large pool of preformed methane, as opposed to methane from slow decomposition of thawing subsea permafrost organic matter, suggests that these releases may be more eruptive in nature, which provides a larger potential for abrupt future releases. The extent to which the source of the methane in the specific seep field at stations 13 and 14 is representative for other documented seepage areas in the Laptev Sea or the ESAS in general, as well as how they are developing over time, remains to be investigated. More triple-isotope data, also temporally resolved, covering a wide range of the inner, mid, and outer shelf in the Laptev, East Siberian, and Chukchi Seas are strongly warranted. Finally, the improved quantitative constraints on the relative importance of different subsea sources in the ESAS and their variability represent a substantial step in our understanding of the system and thus toward credible predictions of how these Arctic methane releases will develop in the future.

## Materials and Methods

### Geophysical Surveying.

Three different echosounder systems were continuously operated during the expedition to guide the selection of detailed study areas and sampling locations: A Chirp subbottom profiler operating between 2.5 and 7.5 kHz for acoustic imaging of the topmost sediment layers beneath the sea floor; a split-beam Simrad EK60 echosounder operated at 18 kHz for qualitative detection of gas bubbles in the water column and to map the distribution of seafloor gas seeps. The typical pulse length ranged from 0.512 to 2.048 ms, operated at either “maximum” or “interval” ping rate. A Kongsberg EM122 12-kHz multibeam system was used for high-resolution bathymetry and water column backscatter analysis. During SWERUS-C3 Leg 1, the typical opening angle of the multibeam varied from 45° to 67° to either side of the centerline. Systematic surveys for seafloor seeps, sites of bubble ebullition in the water column, and other potentially gas-related features in the sediments were conducted in several areas along the cruise track.

### Seawater Sampling.

Samples for CH_4_ concentration and stable-isotope analysis were taken from Niskin-type bottles on a 24-bottle Rosette sampler holding a Seabird 911 CTD equipped with sensors for conductivity (C), temperature (T), depth (D), oxygen, and turbidity, hereafter called "CTD rosette." Water profiles consisted of 8 to 12 samples distributed over a depth range from 5 m below surface to 2 m above the sediments. In addition, horizontal bottom water casts (“drift casts”) were performed for two “hotspot” regions, identified by dense ebullition features on the midwater sonar: positioned upwind from the center of the seep region, the CTD Rosette was then lowered to a few meters above the sediments, and while the ship was drifting slowly over the seep region, bottles were closed sequentially over time to generate a near-bottom horizontal record. Individual bottles were triggered according to the midwater sonar signal before, within, and behind the bubble plume, taking into account the position of the sonar signal relative to the Rosette.

Seawater samples were transferred from the Niskin flasks via silicon tubing to sample containers. We used 60-mL plastic syringes for immediate onboard CH_4_ headspace concentration measurements, and 120-mL glass serum bottles were used to collect samples destined for stable-isotope analysis (two samples for each δ^13^C-CH_4_ and δD-CH_4_ pair). The bottles were filled completely (i.e., no headspace), and samples were preserved with 1 mL of 50% ZnCl_2_ solution and stored at +4 °C until shore-based isotope analysis.

Samples for natural abundance radiocarbon analysis were taken using 60-L Go-Flo bottles (General Oceanics Inc.) for vertical water column profiles and using the seawater intake system for surface waters (8-m intake depth). Water samples were transferred via silicon tubing into 30-L, stainless-steel sample containers (beer kegs “EuroKeg,” Franke Blefa GmbH) fitted with custom-built headpieces including inlet and outlet tubes with one-fourth inch Swagelok quick-connect fittings (see *SI Appendix*, Fig. S5 for more details). Depending on the CH_4_ concentration in the water, two to four kegs were filled. Duplicate samples were taken at the seep stations and single samples at the background stations (as the lower concentrations there required larger volumes of seawater). The kegs were preevacuated and subsequently filled with helium to a pressure of 30 kPar prior to sampling, yielding in a 10-L headspace at ambient pressure after addition of 20 L seawater per keg. Additional control samples for stable-isotope analysis were also taken from the 60-L Go-Flo bottles in 120-mL serum bottles in the same way as from the Niskin bottles to check and eventually correct for any isotope fractionation during extraction and preparation for natural abundance radiocarbon analysis.

### Sample Preparation and Analysis for Dissolved Methane Concentrations.

Headspace equilibration of 40 mL sample water with 15 mL helium was performed directly in the sampling syringe. Immediately after sampling, syringes were equilibrated for 30 min on a shaking table and then left to rest for another 30 min (this resting time has shown to improve the precision of the concentration measurement, as it reduced CH_4_ being in an “undefined state” [i.e., as small bubbles in the water phase]). This method gave reproducible results (relative standard deviation < 4%) that did not differ from headspace analysis using serum bottles. Headspace gas was analyzed on a gas chromatograph (GC) with a flame-ionization detector (GC-FID, Agilent 7890N with PoraBOND column, methanizer, and helium as carrier gas). GC column temperature was adjusted to measure CO_2_ and C_2_H_6_ in addition to CH_4_. Three-point calibration using a helium blank and two standard gases (15 ppm and 150 ppm CH_4_/200 ppm and 2,000 ppm CO_2_/15 ppm and 150 ppm C_2_H_6_, AirLiquide) was performed before and after each station. No ethane was detected at any station and is thus not reported in the manuscript. The concentration of dissolved CH_4_ in the water sample was calculated from the measured headspace concentration using Bunsen solubility coefficients as described in ref. 27.

### Sample Preparation and Analysis for Stable Isotope Composition.

Headspace extraction for stable-isotope analysis was performed after the cruise at Stockholm University. 20 mL of sample water was replaced by a helium headspace, followed by equilibration for 1 h on a shaking table. After a resting time of 30 min (see above), the headspace gas was extracted by replacing it with saturated salt solution and transferred to a smaller (20 mL) vial, prefilled with salt solution to allow for stable storage conditions before analysis. Laboratory tests prior to the expedition showed that no isotopic fractionation occurred in water samples preserved with ZnCl_2_ and stored cold (+4C) for at least 6 mo. Headspace gas from all samples was extracted within that timeframe. For a small subset of samples (*n* = 6), an additional quality check was performed: headspace extractions of triplicates from the same sample were spread out over a time of 120 to 235 d, and δ^13^C-CH_4_ of the samples was measured immediately after extraction. No systematic changes in δ^13^C-CH_4_ or CH_4_ concentration were observed in these samples.

Stable isotope signatures were determined using continuous-flow GC combustion isotope ratio mass spectrometry (GC-C-IRMS). Analysis for δ^13^C-CH_4_ was performed at Stockholm University and analysis for δD-CH_4_ was performed at the Institute for Marine and Atmospheric Research Utrecht University. Both laboratories use an analytical system with a Thermo Finnigan Delta mass spectrometer (Delta V Plus/ Deltaplus XL) and a custom preconcentration device (28, 29). Isotope ratios are reported in the conventional δ notation (30) relative to the Vienna PeeDeeBelemnite and Vienna Standard Mean Ocean Water standards for δ^13^C and δD, respectively. Reproducibility of the measurements (1σ) was 0.3 ‰ for δ^13^C-CH_4_ and 1.4 ‰ for δD-CH_4._

### Sample Preparation and Analysis for Natural Radiocarbon Abundance.

A two-step method modified after that of Kessler and Reeburgh (31) was established for the preparation of large seawater samples for Δ^14^C-CH_4_ analysis by Accelerator Mass Spectrometry (AMS). As a first step, dissolved CH_4_ was extracted from the water samples using custom-built “stripping boards” that were connected to the inlet and outlet tubes of the Eurokeg sample containers within a few hours after sampling. The samples were purged for 2 h with a recirculating helium gas stream, eventually transferring all CH_4_ into the headspace where it was subsequently collected in a cryo-cooled sorbent trap. The sample traps consist of three-eighths inch stainless-steel U tubes, filled with a molecular absorbent (HiSiv3000, one-sixteenth inch pellet form, UOP/Honeywell via Obermeier GmbH). During stripping, traps were immersed into a Dewar with liquid nitrogen (LN_2_) for cooling. The headspace gas was passed through a Drierite/Carbosorb trap to remove water vapor and CO_2_ before the CH_4_ sorbent trap. Sample traps were closed after the stripping process with manual stem needle valves and stainless-steel plugs (Swagelok) and stored at +4 °C until further shore-based processing. The second processing step was performed in the laboratory of Stockholm University, using a dual-stage manifold for CH_4_ purification and conversion to AMS–amenable CO_2_. The sorbent traps were heated to 275 °C to desorb the CH_4_ and concomitant impurities into a recirculating helium gas stream where impurities were oxidized over CuO at 290 °C and removed by cryocondensation at −196 °C (LN_2_). The remaining fraction was recaptured cryogenically in a new sorbent trap (permanently attached to the system) and released into a second loop. CH_4_ was converted to CO_2_ by combustion at 975 °C. The amount of CO_2_ was then quantified by manometry and collected cryogenically in a glass ampoule. The system was checked for potential stable-isotope fractionation using a δ^13^C-CH_4_ standard and processed in the same way as a sample. No isotopic fractionation was detected, and total carbon blanks of the purification system were, on average, 2.7 ± 1.1 µgC. Sample results were corrected for blanks using the amount of carbon manometrically quantified before each subset of samples and the average radiocarbon content determined by AMS on two blanks per sample set. Calculations for blank correction, Δ^14^C-CH_4_ results, and error propagation are shown in *SI Appendix*, text S2. Technical drawings, pictures, and a more detailed description of the system and its operation are displayed in *SI Appendix*, Figs. S5 and S6.

Radiocarbon analyses of the samples were performed at the US-NSF National Ocean Sciences Accelerator Mass Spectrometry facility at the Woods Hole Oceanographic Institution. Radiocarbon values are reported as Δ^14^C and radiocarbon age according to Stuiver and Polach (32).

## Supplementary Material

Supplementary File

## Data Availability

All data used in this study are publicly available at the Stockholm University Bolin Centre for Climate Research Database (https://doi.org/10.17043/swerus-2014-methane-isotopes).
