# Supplementary material for: Source apportionment of methane escaping the subsea permafrost system in the outer Eurasian Arctic Shelf
Source: Proc Natl Acad Sci U S A. 2021 Mar 1;118(10):e2019672118. doi: 10.1073/pnas.2019672118 (PMC7958249; doi:10.1073/pnas.2019672118)
Supplement: Supplementary File [file pnas.2019672118.sapp.pdf]

PNAS
www.pnas.org

Supplementary Information for

Source Apportionment of Methane Escaping the Subsea Permafrost System in the Outer Eurasian Arctic Shelf

Julia Steinbach[1,2,3*], Henry Holmstrand[1,3], Kseniia Shcherbakova[4], Denis Kosmach[4], Volker Brüchert[2,3], Natalia Shakhova[5,6,7], Anatoly Salyuk[4], Célia J. Sapart[8,9], Denis Chernykh[4], Riko Noormets[10], Igor Semiletov[4,5,6], Örjan Gustafsson[1,3*]

[1]*Dept. of Environmental Science, Stockholm University, 106 91 Stockholm, Sweden.*

[2] *Dept. of Geological Sciences, Stockholm University, 106 91 Stockholm, Sweden.*

[3] *Bolin Centre for Climate Research, Stockholm Univ., 106 91 Stockholm, Sweden.*

[4] *V.I. Il'ichev Pacific Oceanological Institute, Far Eastern Branch of the Russian Academy of Sciences, Vladivostok, Russia.*

[5] *Institute of Ecology, Higher School of Economics, Moscow, Russia, Russia.*

[6] *International Arctic Research Center, Univ. of Alaska Fairbanks, Fairbanks, USA.*

[7]*Scientific Centre Moscow State University (MSU)-Geophysics, Moscow, Russia.*

[8] *Laboratoire de Glaciologie, Université Libre de Bruxelles, Brussels, Belgium.*

[9] *Institute for Marine and Atmospheric Research, Utrecht University, Utrecht, The Netherlands.*

[10]*Dept. of Arctic Geology, University Centre Svalbard, Longyearbyen, Norway.*

Corresponding Authors (*):

Julia Steinbach (julia.steinbach@geo.su.se) and

Örjan Gustafsson (orjan.gustafsson@aces.su.se)

This PDF file includes:

Supplementary text (S1-S2)

Figures S1 to S6

Table S1

SI References

SUPPLEMENTARY TEXT

S1: Isotope systematics and endmember pools in ESAS

Stable isotope source signatures ($\delta^{13}C-CH_4$ and $\delta D-CH_4$) reflect the source material and any isotopic fractionation related to the respective methane formation processes, leading to typical $\delta^{13}C$ vs and δD ranges for microbial, thermogenic and abiotic CH_4 as summarized below in Figure S1 *(1,2)*. Observed stable isotopic signatures in nature often originate from a mix of sources; CH_4 from gas hydrates, oceanic seep and mud volcanoes may be mixtures of CH_4 from both microbial and thermogenic origin. Furthermore, isotope signatures can be altered by kinetic fractionation during diffusion and during microbial or abiotic oxidation *(1,3)*. Such fractionation can be useful in deducing the extent of processes such as degradation yet may complicate source tracing (see *(4)* for examples).

Natural abundance radiocarbon composition ($\Delta^{14}C-CH_4$) gives information about the age of the carbon atoms in the CH_4 molecule. Fossil (e.g. thermogenic) sources usually contain no detectable radiocarbon ($\Delta^{14}C-CH_4 < -1000$, corresponding to >60 000 years), whereas contemporary CH_4 production will reflect the age of the substrate e.g. modern plant material or aged peat. The $\Delta^{14}C-CH_4$ is not influenced by fractionation effects (it is by definition corrected by normalizing the isotope ratio measurements to a standard $\delta^{13}C-CH_4$ value). Source interpretations can be, however, difficult if the $\Delta^{14}C-CH_4$ signals reflects a mixture of sources. In addition, recent methanogenesis may use an old (radiocarbon depleted) carbon source.

Our literature review of isotope-based studies in the Siberian Arctic revealed deviations of observed stable isotopic signatures and derived isotopic endmembers from the generic ranges shown in Fig. S1. For example, $\delta^{13}C-CH_4$ values have been observed to be more depleted than usual *(5-7)*. This seems a typical pattern for permafrost regions, as CH_4 originating under low temperatures experiences very slow fractionation during its formation *(4)*. For a more reasonable classification of methane sources to the ESAS bottom waters, the (stable and also radiocarbon) isotopic signatures were grouped into four different endmember pools:

1. **Modern microbial pool:** This represents CH_4 produced in Holocene sediments from the decomposition of terrestrial and marine organic material. This pool also includes

production within the water column where relevant. Expected stable isotope signatures are -120 < $\delta^{13}C-CH_4$< -60 ‰ *(5-7)*, and -350‰ < $\delta D-CH_4$ < -170 ‰ *(1,7)*. Radiocarbon signatures would be similar to those for organic carbon in the ESAS ($\Delta^{14}C-CH_4$ = –232 ±147, *(8,9)*).

2. **Recently produced microbial methane from old substrate**: This represents CH_4 recently formed at the subsea permafrost thaw horizon (at ca 20 - 30 m sediment depth *(10)*). The stable isotope values are the same as for pool 1, - 120 < $\delta^{13}C-CH_4$< -60 ‰ and - 350‰ < δD < -170 ‰ *(4,6,7)*. This methane pool can be distinguished from pool 1 by its significantly greater radiocarbon age, with $\Delta^{14}C-CH_4$ < - 600‰ *(4)*

3. **Old microbial deep pool**: This represents preformed CH_4 held at depth in the ESAS sediment, originally formed as wetlands and Yedoma deposits and then inundated at end of the last glacial period. Methane in this pool can derive either from destabilizing gas hydrates or from rising, subsurface gas. Expected stable isotopic signatures are -120 < $\delta^{13}C-CH_4$< - 60 ‰ and -350‰ < δD < -170 ‰) Radiocarbon signatures would be similar to pool 2 or older.

   Although the isotopic endmember signatures for these three microbial pools can differ from each other locally, depending on exact environmental conditions during formation and exposure time to different alteration processes, the variation within one these pools can be equally large. It is thus not possible to distinguish those pools by their stable isotopic signature alone, thus we have decided to not assign them each distinct signatures, but rather a representative range for all of them.

4. **Thermogenic pool**: This represents CH_4 originating from abiotic production at elevated pressure and temperature. The methane is transported to the sediment surface through bedrock and sediment fractures and talliks. Stable isotopic signatures for thermogenic methane are enriched compared to microbial signatures, with their $\delta^{13}C$ > - 55 ‰ and δD more positive than - 275‰ *(1)*. Signatures in the ESAS are expected to mainly represent natural gas, resulting in ranges of -52 < $\delta^{13}C-CH_4$ < -37

‰ and -210‰ < δD -CH_4< -160 ‰ *(4,11)*. Radiocarbon signatures from this pool have Δ^{14}C-CH_4 = – 1000 ‰ (i.e. no detectable radiocarbon).

S2: Calculations of Δ^{14}C-CH_4 results from raw sample data

Radiocarbon blank characterization and correction

Blank characterization of the Δ^{14}C-CH_4 purification system was performed by attaching an empty sample trap to the system and processing it in the same way as a sample. The size of the blank was determined by manometric quantification that had been calibrated using carbon content measurements from NOSAMS. This procedure was performed before every sample in the beginning, then after a small subset of samples once the blanks became more reproducible. Two blanks (from the beginning and end of the sample progressing period) were sent to NOSAMS for radiocarbon content analysis. The average of that value ($\Delta^{14}C_{blank}$= - 637±108) was used for blank correction of the samples, using the following equation:

$$\Delta^{14}C_{sample} = (\Delta^{14}C_{total} - f_{blank} \cdot \Delta^{14}C_{blank})/f_{sample} \qquad (1)$$

with $\Delta^{14}C_{total}$ being the result reported from NOSAMS, $\Delta^{14}C_{sample} = \Delta^{14}C_{blank\ corrected}$ and $\Delta^{14}C_{blank}$ the respective radiocarbon signals of sample and blank. f_{sample} and f_{blank} are the relative fractions of sample and blank, calculated from the result of the manometric quantification for the uncorrected sample and its proceeding blank ($f_{sample} + f_{blank} = 1$)

Separation of CH_4 and CO_2 contributions to radiocarbon signal

During laboratory preparation of the samples from the SWERUS-C3 2014 expedition, the remaining CO_2 content in the purified CH_4 sample still turned out to be significant, even after two purification steps. The measured radiocarbon content of the combusted sample was thus a mixture CH_4 and CO_2. The residual CH_4 and CO_2 concentrations were quantified by gas chromatography. Duplicate samples allowed us to disentangle the Δ^{14}C signals. For those samples, both Δ^{14}C-CH_4 and Δ^{14}C-CO_2 could be reported. These data were then used to calculate Δ^{14}C-CH_4 for the remaining (single) samples, as Δ^{14}C-CO_2 showed no strong variations or trends (see table S1).

The total radiocarbon content of a sample containing CH_4 and CO_2 is determined as:

$$\Delta^{14}C_{total} = \Delta^{14}C - CH_4 \cdot f_{CH4} + \Delta^{14}C - CO_2 \cdot f_{CO2} \quad (2)$$

For a set of duplicates for one sample we get therefore

$$\Delta^{14}C_{total,1} = \Delta^{14}C - CH_4 \cdot f_{CH4,1} + \Delta^{14}C - CO_2 \cdot f_{CO2,1} \quad (3)$$

$$\Delta^{14}C_{total,2} = \Delta^{14}C - CH_4 \cdot f_{CH4,2} + \Delta^{14}C - CO_2 \cdot f_{CO2,2} \quad (4)$$

These equations are solved for Δ^{14}C-CH_4 and Δ^{14}C-CO_2:

$$\Delta^{14}C - CO_2 = \frac{(f_{CH4,1} \cdot \Delta^{14}C_{total,2} - f_{CH4,2} \cdot \Delta^{14}C_{total,1})}{f_{CH4,1} \cdot f_{CO2,2} - f_{CH4,2} \cdot f_{CO2,1}} \quad (5)$$

$$\Delta^{14}C - CH_4 = \left(\Delta^{14}C_{total,1} - \Delta^{14}C - CO_2 \cdot f_{CO2,1}\right)/f_{CH4,1} \quad (6)$$

The relative contributions of CH_4 and CO_2 in the sample are determined from their mixing ratios ([CH_4] and [CO_2]), that were gas-chromatically measured before combustion of the sample:

$$f_{CO2} = [CO_2]/([CO_2] + [CH_4]) \quad (7)$$

$$f_{CH4} = [CH_4]/([CO_2] + [CH_4]) \quad (8)$$

For calculation of Δ^{14}C-CH_4 from samples without duplicate, Δ^{14}C-CO_2 in equation (6) was set to the average value of Δ^{14}C-CO_2 derived from the duplicate samples (Δ^{14}C-CO_2 = –976 ± 29). The value showed no significant variation between stations and throughout the water column (see Table S1).

Error assessment

The error of the disentangled Δ^{14}C-CH_4 signals is calculated using error propagation:

$$err(\Delta^{14}C - CH_4) = \frac{\partial(\Delta^{14}C - CH_4)}{\partial\left(\Delta^{14}C_{total,1}\right)} \cdot err\left(\Delta^{14}C_{total,1}\right) + \frac{\partial(\Delta^{14}C - CH_4)}{\partial(\Delta^{14}C - CO_2)} \cdot err(\Delta^{14}C - CO_2)$$

$$+\frac{\partial(\Delta^{14}C - CH_4)}{\partial(f_{CO2,1})} \cdot err\left(f_{CO2,1}\right) + \frac{\partial(\Delta^{14}C - CH_4)}{\partial(f_{CH4,1})} \cdot err\left(f_{CH4,1}\right)$$

$$\frac{err(\Delta^{14}C_{total,1})}{f_{CH4,1}} + \frac{f_{CO2,1}}{f_{CH4,1}} \cdot err(\Delta^{14}C - CO_2) \cdot + \frac{\Delta^{14}C - CO_2}{f_{CH4,1}} \cdot err(f_{CO2,1})$$

$$\frac{+\Delta^{14}C - CO_2}{{f_{CH4,1}}^2} \cdot err(\Delta f_{CH4,1}) \qquad (9)$$

with $\frac{\partial(\Delta^{14}C - CH_4)}{\partial(x)}$ the partial derivatives of the contributing variable x with error *err(x)*.

The separate errors are calculated as follows:

$$err(\Delta^{14}C_{total}) = err(\Delta^{14}C_{raw}) + \frac{f_{blank}}{f_{sample}} err(\Delta^{14}C_{blank}) \qquad (10)$$

with $err(\Delta^{14}C_{raw})$ the analytical error given by NOSAMS and $err(\Delta^{14}C_{blank})$ the error of the blank average.

For duplicates, $err(\Delta|14|C - CO_2)$ is given by

$$err(\Delta^{14}C - CO_2) = \frac{(f_{CH4,1} \cdot err(\Delta^{14}C_{total,2}) + f_{CH4,2} \cdot err(\Delta^{14}C_{total,1})}{abs(f_{CH4,1} \cdot f_{CO2,2} - f_{CH4,2} \cdot f_{CO2,1})} + err(f_{CH4}, f_{CO2}) \qquad (11)$$

As the errors in the CH_4 and CO_2 fractions are connected to each other, their combined error was calculating by using the span of $\Delta^{14}C$-CO_2 calculated with a realistic error in each fraction (1%). For single samples, $err(\Delta^{14}C - CO_2)$ is given by the error in the average $\Delta^{14}C$-CO_2 value derived from the duplicate samples.

SUPPLEMENTARY FIGURES

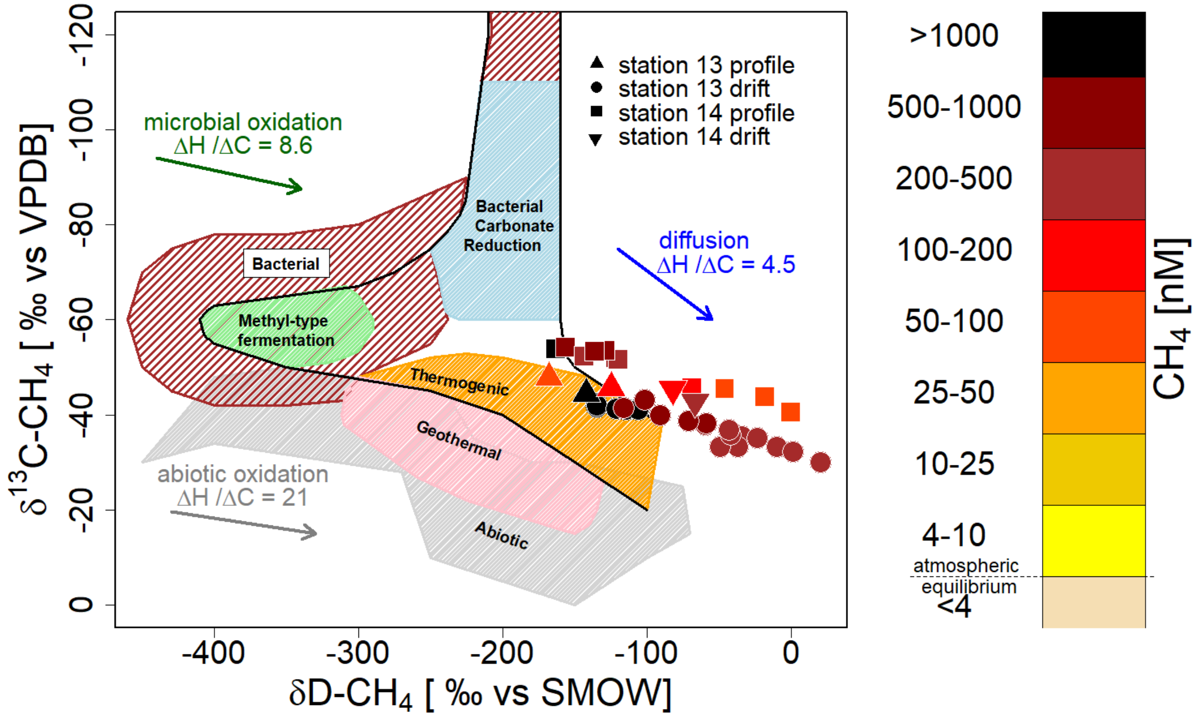

Figure S1: Classification of stable isotope signatures of methane sources and pathways

Stable isotope data from vertical and horizontal profiles at the two main seep stations are shown in the context of typical literature-based isotope classification ranges. Isotopic ranges for methane formation pathways are based on *(1,2,12).* Isotopic fractionation slopes are added for microbial oxidation *(3, 13)*, abiogenic oxidation *(14)*, and diffusion *(15).* Data from station 13 and 14 are coloured according to their dissolved methane concentration, showing clearly a deviation from the “classical” ranges for lower concentrations that correspond to samples taken further away from the seep source.

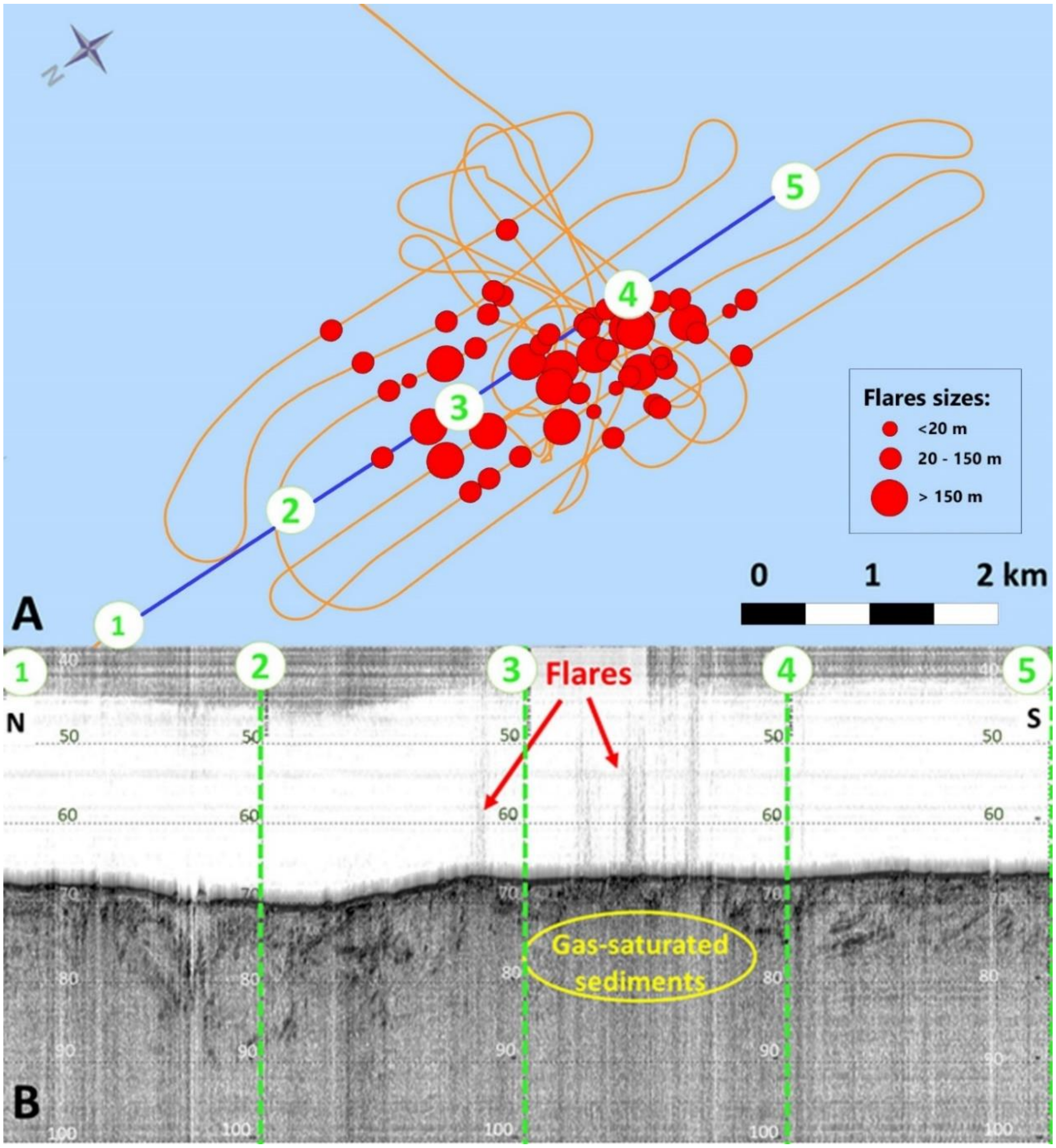

Figure S2: Subbottom profile from station 13

Panel (A) is showing the location of the profile within the seep field, Panel (B) the profile data. The locations of the numbered dots on the map correspond to numbers on the profile picture. The profile shows clearly the “structureless” sediments under the seafloor gas flares (mostly between the points 3 and 4), indicating gas-saturated sediments that “kill” the acoustic signal and do not allow its penetration deeper into the sediments. In contrast, the acoustic signals on either side of the saturated sediments are able to penetrate much deeper, revealing various structures (layering) in the sediments.

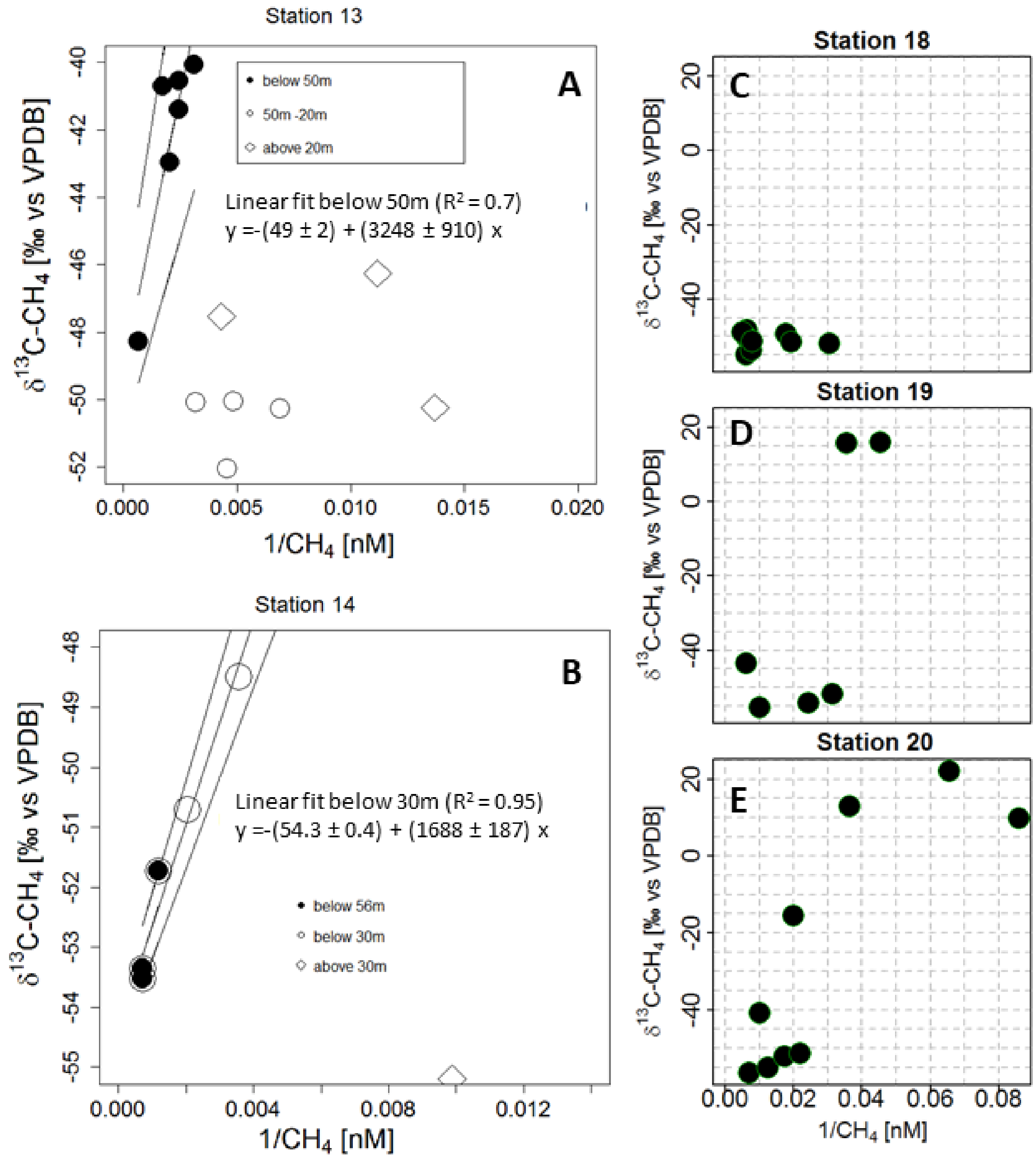

Figure S3: **Keeling plots for vertical water column samples**

Illustration of inconclusive Keeling plots for mid-water and surface samples at station 13 (A) and 14 (B), as well as for remaining sampling locations located further away from the strongest seeps (C – E).

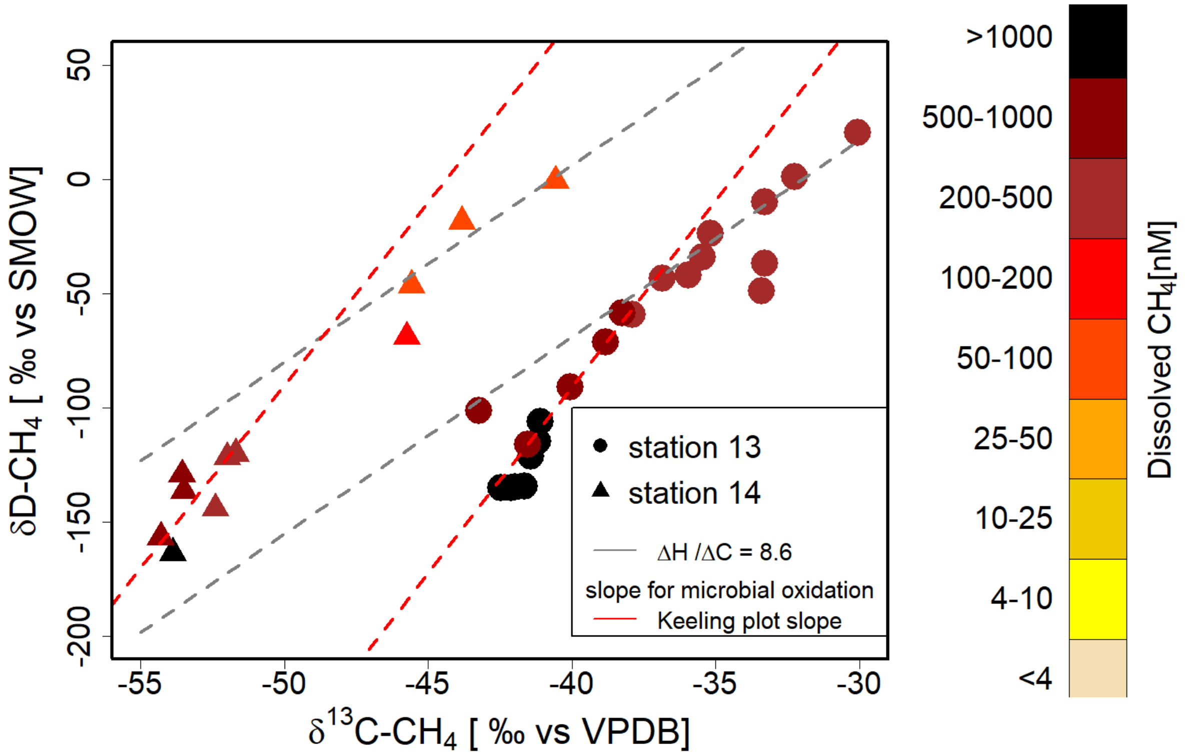

Figure S4: **Isotopic slopes close to and further away from the seep centers**

δD-CH_4 vs δ^{13}C-CH_4 for drift stations 13 and 14, with the colors representing methane concentration as displayed in the legend. The plot clearly illustrates a difference in the δD-CH_4/ δ^{13}C-CH_4 slope between samples with lower concentration (= further away from the seep center) and those with higher concentrations (=closer to the seep center). The grey lines show a slope of ΔH/ΔC=8.6 typical for microbial oxidation *(3,13)*, which describes well the data further away from the seeps. A steeper slope results from the linear fit for the high-concentration samples (red line, result of Keeling plots shown in Figure 4 in the main text). This slope is instead dominated by mixing of the seep source(s) and the (fractionated) background.

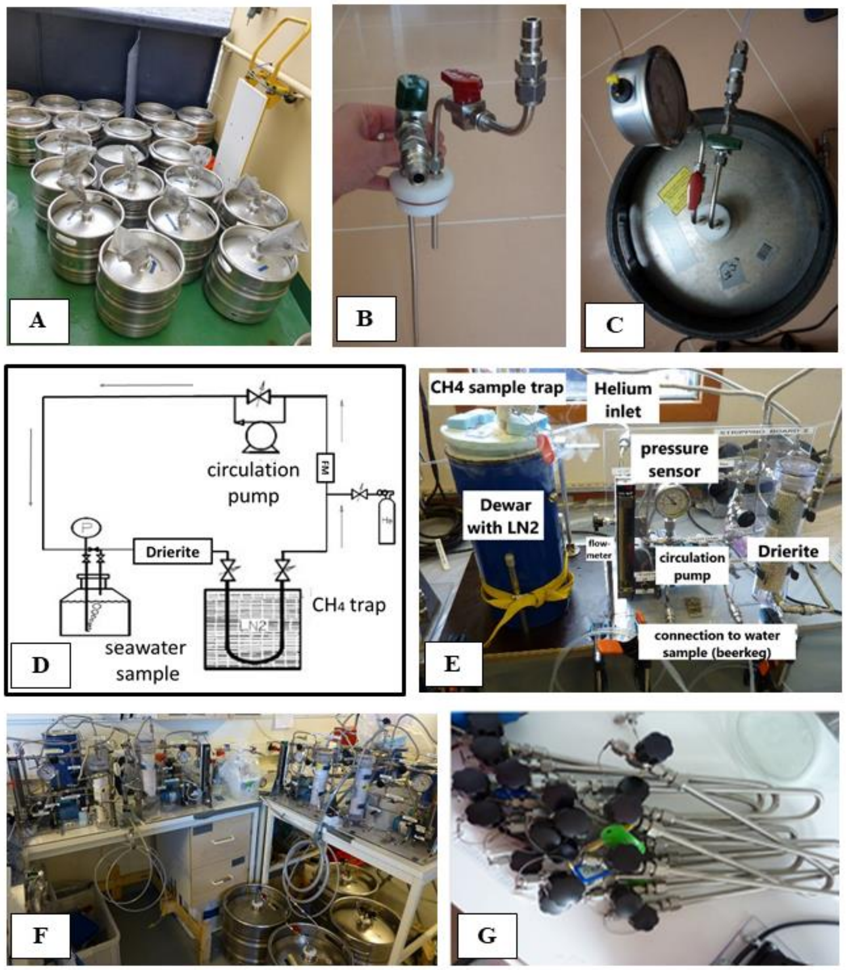

Figure S5. Drawings and illustrations for shipboard Δ^{14}C-CH4 sample processing system

Panel (A) shows sample kegs prepared for subsampling of GO-FLO bottles onboard I/B Oden and Panel (B) custom built headpiece with tubes, shut-off valves and quick connects. The long tube goes down to the bottom of the keg and is used for filling the keg during sampling and as inlet for Helium purging during stripping. The short S-shaped tube ends in the headspace. During stripping, it forms the outlet to the stripping board; during keg filling it connects to a pressure sensor for monitoring headspace pressure = filling status. Panel (C) shows the assembled keg with headpiece in sampling configuration (pressure sensor and inlet tube connected). Panels (D) and (E) show flow scheme and illustrative picture of the stripping board. Panel (F) illustrates the setup of the four stripping boards as used during the SWERUS-C3 expedition, with kegs for stripping standing below on the floor. Panel (G) shows sorbent traps for CH_4 samples.

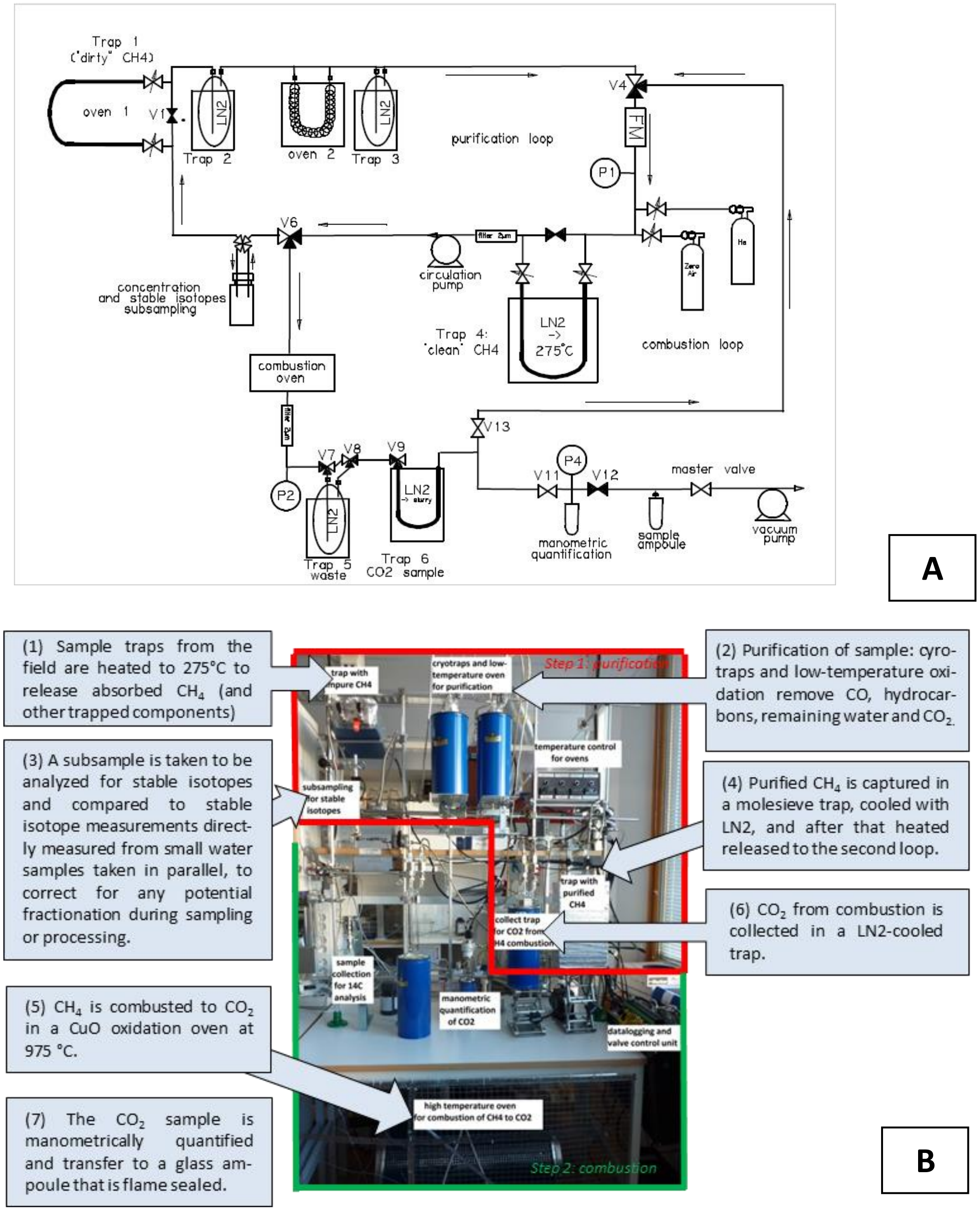

Figure S6. Illustrations for laboratory Δ^{14}C-CH4 sample processing system

Panel (A) shows the flow scheme of the purification-combustion system and Panel (B) the laboratory setup of the system at Stockholm University and description of the different processing steps. The system operates semi-automatically: While datalogging and switching of valves is controlled by a CR1000 datalogger (Campbell Scientific), the operator has to start the different steps on graphical computer interface (RMTC RunTime) and to perform a few manual tasks (e.g. adding LN_2 Dewars, flame sealing).

SUPPLEMENTARY TABLE

Table S1: $\Delta^{14}C$-CH_4 results. Summary of sample results for natural radiocarbon abundance of CH_4, documenting the step from raw data to final result using equation (1) - (11) from the section S2. Duplicate samples are displayed in green and single samples in yellow.

Sample information				Quality information*		Analytical results (ACES/NOSAMS)					results calculated following equations (1) - (11)					
Station	depth [m]	Sample ID	Paired samples (same character marks pairs)	Fraction blank < 0.3 ?	Existing duplicate of sufficient quality?	Carbon content in sample [µg C]	Measurement blank previous to sampling [µg C]	Blank contribution [%]	$[CH_4/CO_2]$ mixing ratios	Δ14C of sample (NOSAMS analysis)	Δ14C of sample (blank-corrected)	Forced Δ14C-CO_2 for samples w/o duplicate	Δ14C in CO_2 fraction	Δ14C in CH_4 fraction	CO_2 age [kyr]	CH_4 age [kyr]
13	20	STN13_GoFLO_V_20m	b	yes	yes	23	0.6	9	26.34	-744 +/- 8	-755 +/- 17	-	-910	-749 +/- 29	17.8 - 21.1	10.2 - 12.1
13	20	STN13_GoFlo_VI_20m	b	yes	yes	60	4.3	10	0.09	-871 +/- 2	-897 +/- 13	-				
13	50	STN13_GoFLO_IV_50m	c	yes	yes	71	3.7	7	1.31	-911 +/- 1	-933 +/- 9	-	-1051	-842 +/- 73	>60 (radiocarbon dead)	11.7 - 19.8
13	50	STN13_GoFlo_III_50m	c	yes	yes	40	4.2	14	0.27	-955 +/- 3	-1007 +/- 18	-				
13	60	STN13_GoFlo_II_60m	d	yes	yes	194	4.3	3	1.11	-968 +/- 1	-979 +/- 4	-	-1012	-949 +/- 28	32 - (> 60)	20.3 - 30.2
13	60	STN13_GoFlo_I_60m	d	yes	yes	337	1.6	1	3.33	-960 +/-1	-964 +/- 2	-				
14	59	STN14_GoFlo_I_59m	f	yes	yes	66	1.6	5	0.19	-936 +/- 2	-952 +/- 8	-	-930	-1067+/- 65	19.5 - 23.7	>60 (radiocarbon dead)
14	59	STN14_GoFlo_II_59m	f	yes	yes	189	2.6	2	0.78	-982 +/- 1	-990 +/- 3	-				
14	25	STN14_GoFlo_IV_25m	-	yes	no	82	2.6	5	0.14	-940 +/- 2	-956 +/- 7	-976 +/- 29	-	-815 +/- 417	-	4.1 - (>60)
14	8	STN14_SWI_II_13.20	-	yes	no	25	2.4	15	0.16	-920 +/- 5	-970 +/- 27	-976 +/- 29	-	-933 +/- 507	-	4.4 - (>60)
19	8	STN19-SWI_21:45_I	-	yes	no	18	2.4	20	10.53	-545 +/- 4	-521 +/- 20	-976 +/- 29	-	-478 +/- 36	-	4.6 - 5.8

*Sample results with a blank/signal fraction >0.3 were considered insufficient quality and excluded from further processing and discussion in this manuscript.

SI References

1. M.J. Whiticar, Carbon and hydrogen isotope systematics of bacterial formation and oxidation of methane. *Chem. Geol.* **161**(1–3), 291–314 (1999)

2. J.R. Melton, M. J. Whiticar, P. Eby, Stable carbon isotope ratio analyses on trace methane from ice samples. *Chem. Geol.* **288** (3–4), 88–96 (2011).

3. D.D. Coleman, J.B. Risatti, M. Schoell, Fractionation of carbon and hydrogen isotopes by methane-oxidizing bacteria. *Geochim. Cosmochim. Acta*, **45**(7), 1033–1037 (1981).

4. N. Shakhova, I. Semiletov, E. Chuvilin. Understanding the Permafrost–Hydrate System and Associated Methane Releases in the East Siberian Arctic Shelf. *Geosciences **9***, 251 (2019).

5. C. J. Sapart, N. Shakhova, I. Semiletov, J. Jansen, S. Szidat, D. Kosmach, O. Dudarev, C. van der Veen, M. Egger, V. Sergienko, A. Salyuk, V. Tumskoy, J.-L. Tison, T. Röckmann, The origin of methane in the East Siberian Arctic Shelf unraveled with triple isotope analysis. *Biogeosciences* **14**, 2283–2292 (2017).

6. E.M. Galimov, Isotope organic geochemistry. *Org. Geochem.* **37**(10), 1200-1262 (2006).

7. E.M. Rivkina, G. N. Kraev, K. V. Krivushin, K. S. Laurinavichus, D. G. Fyodorov-Davidov, A. L. Kholodov, V. A. Scherbakova, D. A. Gilichinsky, Methane in permafrost of northeastern Arctic. *Cryosphere of the Earth* **10**, 23– 41 (2006)

8. J.E. Vonk, L. Sanchez-Garcia, B. van Dongen, V. Alling, D. Kosmach, A. Charkin, I. Semiletov, O. Dudarev, N. Shakhova, P. Roos, T. Eglinton, A. Andersson, Ö. Gustafsson, Activation of Pleistocene carbon by erosion of coastal permafrost in Arctic Siberia. *Nature* **489** (7414), 137-40 (2012).

9. T. Tesi, I. Semiletov, O. Dudarev, A. Andersson, Ö. Gustafsson, Matrix association effects on hydrodynamic sorting and degradation of terrestrial organic matter during cross-shelf transport in the Laptev and East Siberian shelf seas. *J. Geophys. Res. Biogeosci.* **121**, 731– 752 (2016).

10. D. Nicolsky, N. Shakhova, Modeling sub-sea permafrost in the East Siberian Arctic Shelf: The Dmitry Laptev Strait. *Environ. Res. Lett.* **5**(1), 015006 (2010).

11. V.A. Istomin, Gas hydrates in the permafrost zone. *Gas Ind. Russ.* **4**, 16–27 (2006).

12. G. Etiope, M Schoell, Abiotic gas: atypical, but not rare. *Elements* **10**(4), 291–296 (2014).

13. F. S. Kinnaman, D. L. Valentine, S. C. Tyler, Carbon and hydrogen isotope fractionation associated with the aerobic microbial oxidation of methane, ethane, propane and butane. *Geochim. et Cosmochim. Acta* **71**, 2, 271-283 (2007).

14. G. Etiope., C. Baciu, M. Schoell, Extreme methane deuterium, nitrogen and helium enrichment in natural gas from the Homorod seep (Romania). *Chem. Geol.* **280**, 89–96 (2011).

15. M. Schoell, Genetic characterization of natural gases, *Am. Assoc. Pet. Geol. Bull.*, **67**, 2225–2238 (1983).
